# Resource-Strengthening Training for Parents of Adolescents with Problematic Gaming (Res@t-P): A Clinical Pilot Study

**DOI:** 10.3390/ijerph19159495

**Published:** 2022-08-02

**Authors:** Joel Hülquist, Nicole Fangerau, Rainer Thomasius, Kerstin Paschke

**Affiliations:** German Center for Addiction Research in Childhood and Adolescence (DZSKJ), University Medical Center Hamburg-Eppendorf (UKE), Martinistraße 52, 20246 Hamburg, Germany; joel.huelquist@stud.uke.uni-hamburg.de (J.H.); n.fangerau@uke.de (N.F.); thomasius@uke.de (R.T.)

**Keywords:** gaming disorder, family factors, parental factors, adolescents, intervention study, group therapy

## Abstract

*Background*: Problematic gaming (PG) has become an increasing mental health issue among adolescents during the preceding years. The role of parents and the family environment in the development of PG has been repeatedly emphasized. However, the structured involvement of parents in the therapy is still largely insufficient. Resource-strengthening training for parents of adolescents with PG (Res@t-P) is a new parent-centered 8-week group intervention to fill this substantial gap. The present pilot study aimed to collect first information on its potential effectiveness in improving parental and family factors. *Methods*: The study was conducted in a clinical setting with N = 43 parents of adolescents with PG, applying a pre- and post-follow-up design. Standardized questionnaires on psychological stress perception, family communication, family functioning, media rules, and adolescent PG symptoms were applied at three measurement points (before, at the end of, and 6 weeks after the training). Conditional growth models were estimated. *Results*: Over time, an improvement in parental and family aspects as well as a reduction in adolescent PG symptoms could be observed. *Conclusions*: The results of the present pilot study on the effectiveness of Res@t-P are promising. No causal inferences can be drawn at this stage. A randomized-controlled intervention study is highly warranted.

## 1. Introduction

Everyday life is increasingly shaped by digital media and rising screen time. Usage times of digital games have significantly risen in adolescents under the COVID-19 pandemic [1,2]. For adolescents with specific risk factors, intensive use can lead to addictive gaming patterns, resulting in significant personal, family, social, academic, and occupational problems, putting a burden on healthcare systems and economics [3,4,5]. Globally, the prevalence for problematic gaming (PG) is estimated between 3.1% and 3.3%, with a special emphasis on the period of adolescence [6,7].

The first criteria for PG were described under the term *Internet Gaming Disorder* (IGD) in the appendix of the *Diagnostic and Statistical Manual of Mental Disorders* (DSM-5) in 2013 as a condition requiring further research [8]. Five out of nine criteria have to be fulfilled over a period of 12 months for it to be considered an IGD. These include: (1) preoccupation with gaming, (2) withdrawal, (3) tolerance, (4) unsuccessful attempts to reduce or stop gaming, (5) giving up other activities, (6) continuation of gaming despite problems, (7) deceiving or covering up gaming, (8) gaming to escape adverse moods, and (9) risking or losing relationships or career opportunities due to gaming. To underline the clinical significance of this new phenomenon, *Gaming Disorder* (GD) was included as an official diagnosis in the 11th version of the International Classification of Diseases (ICD-11) by the World Health Organization in 2018 [9]. Accordingly, a GD is assumed when the following criteria with regard to gaming are met: (1) loss of control, (2) increased prioritization, and (3) continuation despite negative consequences. The criteria should be present for at least 12 months in general and lead to significant impairments in personal, educational, and social life. In addition, the risk for potentially developing GD is indicated by the term *Hazardous Gaming*. ICD-11 GD criteria differ from DSM-5 IGD criteria by their higher diagnostic threshold since besides specific symptoms, significant impairments arising from them must be present [10,11,12,13,14]. To account for both definitions, the term *PG* will be used as an umbrella term in this study.

The etiology of PG is multifactorial and based on biological, psychological, parental, family, peer, and society risk factors [4,15,16,17]. Interactional aspects between personal, parental, family, and peer factors are emphasized in the Interactional Theory of Childhood Problematic Media Use (IT-CPU) [18]. Accordingly, parents and family members play a key role in the development and maintenance of PG, including their own media usage behavior. Moreover, an influence of parent-child relationship, marital status, parental and family communication, parenting and rule behavior, parental health, as well as family functioning on PG etiology could be shown [19,20,21,22,23].

Therapeutic approaches include pharmacological therapy of comorbidities, virtual reality, and cognitive-behavioral therapy (CBT), with the latter showing the best effects on PG symptom reduction [24,25]. To date, there is still a lack of evidence-based treatment programs, especially for adolescents [26]. Moreover, therapeutic interventions usually address the affected adolescent in group or individual settings and, thus, mainly focus on intra-individual rather than interindividual factors [27]. Family factors are accounted for only by some programs, e.g., [28] and parents’ involvement is mostly limited, e.g., to psychoeducation [29]. By applying individual, group, and family sessions, these programs show a significant improvement in PG symptoms but no additional benefit over classical CBT [28,29]. In contrast, multidimensional family therapy (MDTF) addresses individual personal, parental, family, and environmental factors in a manualized setting and could be shown to be superior to classical family therapy in the reduction of PG symptoms in a randomized-controlled trial (RCT) study with a small sample size [30]. A recent meta-ana-lysis of Asian intervention studies could not find a benefit of the involvement of parents in the efficacy of PG treatment [31]. In contrast, another systematic review highlighted the importance of parental support and involvement for treatment success [27]. However, given the limited parental involvement in therapy programs in general, treatment and study heterogeneity, the lack of RCT studies, low sample numbers, high drop-out rates, and differences not only between cultures but also within underlying PG concepts and measures, a final conclusion is questionable at this point [26].

Epidemiological research highly supports the assumption that parental and family factors need to be addressed more specifically [32]. Furthermore, clinical experts call for the targeted involvement of affected parents [33] to address parental needs, parenting behavior [34], their important function as role models [35], and also mental health aspects [36] and the distress associated with the PG of their child [37].

The current study aims to reduce this significant gap by introducing the newly developed and first manualized group-therapy program for parents of adolescents with PG (resource-strengthening training for parents of adolescents with PG, Res@t-P) and investigating its efficacy in improving parental and family factors within a pilot-study design.

## 2. Materials and Methods

### 2.1. Res@t-P

Res@t-P is CTB-based group training with elements of mindfulness, pedagogical, developmental, and communication psychology for parents whose children have been diagnosed with PG. It is part of the Res@t program that, in addition, involves manualized group treatment for the affected adolescents themselves (resource strengthening training for adolescents with PG, Res@t-A). Adolescents are referred to as those aged 10 to 19 years based on the WHO definition. Both programs should be applied in parallel. They were developed by experienced practitioners and researchers specialized in behavioral addictions at the German Center for Addiction Research in Childhood and Adolescence (DZSKJ) at the University Medical Center Hamburg-Eppendorf (UKE).

Both training programs are composed of eight modules spread over the duration of 8 weeks that are preceded by an individual family session with the group therapist to provide program information and strengthen therapy coherence. Additionally, a midterm and a final program evaluation with the individual family is planned. Six to eight weeks after the last module, a booster session within both groups is scheduled separately. Res@t is designed for the in- and outpatient setting with a partially open-group structure to meet clinical demands, i.e., entry into the program is possible at four predefined time points.

**Figure 1:**
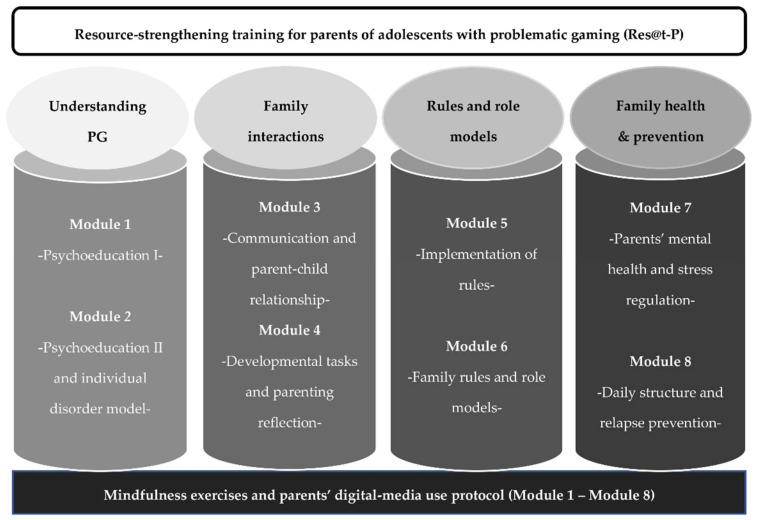
Thematic pillars of the training and content overview. Notes: PG = problematic gaming.

Each Res@t-P module lasts 90 min, and the group size should be limited to 10 parents. The program is built on four thematic pillars (Figure 1). These are psychoeducation about PG, including etiology and risk factors, diagnosis, and the development of a specific disorder model for the individual child (modules 1 and 2); communication techniques (including validation and invalidation, as well as I and you messages), children’s developmental tasks, and associated parenting challenges with parenting reflection (modules 3 and 4); the implementation of rules based on reinforcement learning and family counseling as well as parents’ different roles and modeling (modules 5 and 6); and reflection on parents’ mental health, psychological stress perception, and regulation as well as daily structuring, adolescents’ alternative activities, awareness of risk situations, and creation of an emergency kit to prevent relapse (modules 7 and 8). Parents are invited to keep a record of their own digital media use via a smartphone tracking app to increase self-awareness. Each module starts with a mindfulness exercise and an individual reflection of the past week experiences, including media use. The interaction between parents is encouraged throughout the training to facilitate sharing experiences and group support.

### 2.2. Participants and Procedure

N = 43 parents of adolescents with diagnosed PG participated in the study between November 2019 and August 2021, either alone or with the partner parent (Table 1). Diagnosis was undertaken by child and adolescent psychiatrists and psychotherapists experienced in the field of behavioral addictions based on the DSM-5 and ICD-11 criteria of PG. The corresponding adolescents were treated in an outpatient or (partial) inpatient setting at the addiction-specialized child and adolescent psychiatry and psychotherapy section of the UKE. Thus, in addition to Res@t-P, family sessions took place (up to every second week) and adolescents received individual and group therapy offers as treatment as usual.

In all, 8 parents did not finish the program and could be assessed only at the beginning of the training. Reasons were job (three parents), stress (two parents), and COVID-19 related (two parents) or included a lack of coherence (one parent). Thus, the dropout rate was 18.6%. The remaining 34 parents filled out questionnaires at three measurement points (at the beginning of the training, at the end of the training after 8 weeks, and 6 weeks after the last session).

The study followed the ethical guidelines of the relevant national and institutional committees on human experimentation, was in accordance with the Declaration of Helsinki, and was approved by the Local Psychological Ethics Commission at the Center for Psychosocial Medicine (LPEK) of the UKE (LPEK-0053, approved 25 July 2019). Each parent gave his or her informed consent prior to participation and could withdraw from the study at any time for any reason. Data were pseudonymized and stored in the protected data room of the UKE.

### 2.3. Measures

#### 2.3.1. Psychological Factors

Psychological stress perception of the parents was assessed using the 4-item version of the Perceived Stress Scale (PSS-4) [38]. This questionnaire was validated in large international samples [39] and asks how frequently subjects have appraised their life as unpredictable, uncontrollable, and overloading within the previous month. Total scores of the Likert-scaled items range between 0 and 16, with higher scores indicating more stress perception and values ≥ 8 being referred to as elevated [40].

In addition, psychological distress was measured with the established Brief Symptom Inventory (BSI-18) to measure the baseline condition [41]. Three subscales (somatization, depressiveness, and anxiety) form 18 Likert-scaled items, summing up to a maximum score of 72, and higher scores indicate higher distress [42].

#### 2.3.2. Parenting Factors

Parental self-confidence was assessed by the Likert-scale 15-item Parental Self-Efficacy Questionnaire (FSW), an expanded version of the widely used 9-item Parenting Self-Efficacy Questionnaire [43]. A maximum score of 60 points can be achieved with higher scores being associated with more efficacy.

The implementation of rules associated with the adolescent’s use of digital media (including quantities, settings, and reliability) was assessed by the 6-item Likert-scaled Media Rules Questionnaire (MR-6). Higher scores indicate a more appropriate implementation of rules and sum up to 18 points maximum. MR-6 was earlier used in a large representative study with 1221 parents, where it showed good internal consistency (Cronbach’s ⍺ = 0.84) [44].

#### 2.3.3. Family Factors

The quality of communication between parents and children was obtained with the Family Communication Scale (FCS) of the validated Family Adaptability and Cohesion Evaluation Scale IV (FACES IV) [45,46]. Higher scores indicate more sophisticated communication. Scores of the 10 Likert-scaled items can be added up to 50 points maximum.

Family functioning was measured with the validated short version of the German Family Questionnaire (*Familienbogen-Kurzversion*, FB-K) [47]. A maximum score of 60 can be achieved by 20 Likert-scaled items, suggesting optimal functioning.

#### 2.3.4. Adolescent PG

The number of adolescent PG symptoms was rated by the parents with the Parental Internet Gaming Disorder Scale (PIGDS) [48]. This 9-item questionnaire is based on the DSM-5 criteria of IGD using a binary response pattern (yes = 1/no = 0), with each yes answer resembling one fulfilled criterion. The cut-off for IGD is reached with a total score of ≥5.

### 2.4. Statistical Analysis

All statistics were performed with the software package R version 4.0.3 (R Foundation for Statistical Computing, Vienna, Austria) [49].

#### 2.4.1. Data Management

Response patterns on standardized scales with missing responses to single-item measures were replaced by a multiple imputation procedure (package mice, [50]). Data were revised for normality distribution if appropriate, with absolute values of skewness > 2.0 and kurtosis > 7.0 indicating substantial non-normality [51].

#### 2.4.2. Conditional Growth Models

Psychological, parenting, and family factors as well as adolescent PG were included as criterion variables in six conditional growth models with random slopes and intercepts for individual trajectories [52] using the function lmer of R package lme4 [53]. In addition to nesting individuals over the three measurement points, nesting of parents when participating as a pair took place to account for data dependence. The number of attended sessions and the parental distress (BSI-18 score) at the start of the training were included as covariates to control for potential outcome confounders. Scale scores were z-scaled for easier interpretation of model parameters. Incidence rate ratios (ICC) were calculated with 95% confidence intervals (CIs). Model parameter degrees of freedom and *p*-values were estimated based on Wald t-distribution approximation. A Bonferroni corrected *p*-value p_bonf_ ≤ 0.00833 indicates significance within each of the six models to account for potential Type-I errors due to multiple testing.

## 3. Results

### 3.1. Sample Characteristics

Demographic characteristics as well as sum scores of psychometric measures are presented in Table 1.

### 3.2. Psychological Factors

**Figure 2:**
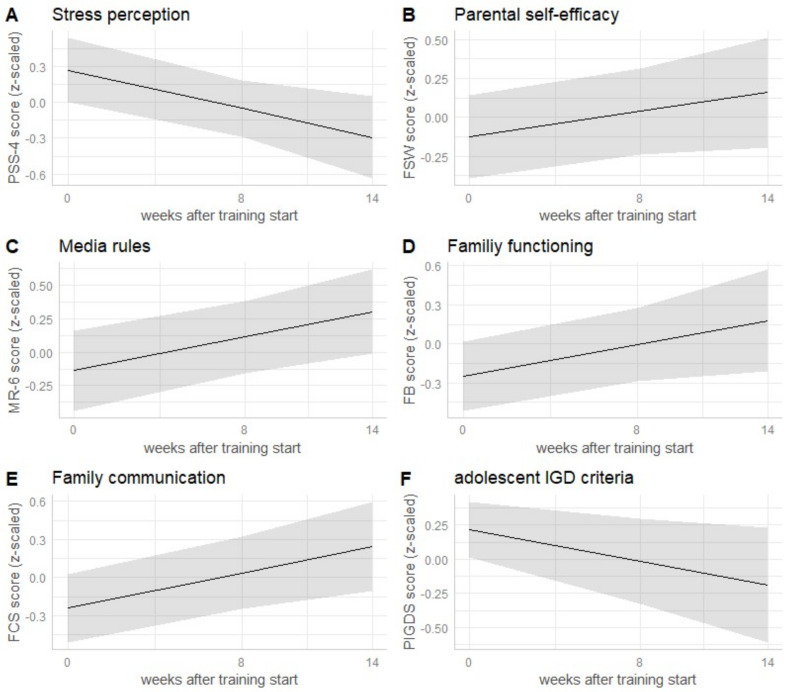
Latent growth models on six z-scaled criterion variables over weeks of study. Data were acquired before the start of the training (week 0), at the end of the training (week 8), and after a 6-week follow-up (week 14). Regression line is shown with a 95% CI. Notes: PSS-4 = Perceived Stress Scale (4-item version); FSW = Parental Self-Efficacy Questionnaire; MR-6 = Media Rules Questionnaire (6 items); FCS = Family Communication Scale; FB = Family Questionnaire (*Familienbogen-Kurzversion*); PIGDS = Parental Internet Gaming Disorder Scale.

Psychological stress perception of the parents decreased over the three measurement time points, with an IRR of 0.96 per week. The number of attended sessions and especially parental distress were shown to be covariates that should be considered, both with a positive association with PSS-4 scores. Time explained 22.0% of the model variance (Table 2; Figure 2A).

### 3.3. Parenting Factors

An increase in parental self-efficacy could be observed on a descriptive level but not significantly over the time course in the conditional growth model. Parental distress was negatively associated with FSW scores in the model as a covariate with an IRR of 0.76 per week. Time explained 8.5% of the model variance (Table 2; Figure 2B). Over time, more media rules were implemented by the parents, with an IRR of 1.03 per week. Time explained 9.4% of the model variance (Table 2; Figure 2C).

### 3.4. Family Factors

Family functioning increased over the course of the training and follow-up, with an IRR of 1.03 per week. Time explained 4% of the model variance (Table 2, Figure 2D). Moreover, the quality of communication between the parents and their children improved over time, with an IRR of 1.04 per week. Time explained 9.9% of the model variance (Table 2; Figure 2E).

### 3.5. Adolescent PG

The number of adolescent PG symptoms slightly decreased over time, with an IRR of 0.97 per week, and the number of attended sessions was identified as a significant covariate with an inverse association. Time explained 13.7% of the model variance (Table 2; Figure 2F).

**Table 2:** Conditional growth models.

	Predictors	IRR	CI	*p*	t(df)
**Psychological factor**					
	(Intercept)	0.52	0.23–1.19	0.122	−1.56 (87)
	Week	**0.96**	0.93–0.99	0.009	−2.66 (87)
**PSS-4**	Covariates				
	Attended sessions	**1.15**	1.01–1.31	0.036	2.13 (87)
	BSI-18	**1.44**	1.15–1.81	**0.002**	3.25 (87)
	*R*^2^*_marg_ =* 0.220		*R*^2^*_cond_ =* 0.701		
**Parental factors**					
	(Intercept)	1.13	0.48–2.67	0.775	0.29 (94)
	Week	1.02	1.00–1.04	0.083	1.75 (94)
**FSW**	Covariates				
	Attended sessions	0.96	0.84–1.10	0.575	−0.56 (94)
	BSI-18	**0.76**	0.59–0.98	0.036	−2.13 (94)
	*R*^2^*_marg_ =* 0.085		*R*^2^*_cond_ =* 0.633		
	(Intercept)	0.41	0.16–1.05	0.064	−1.87 (91)
	Week	**1.03**	1.01–1.05	**0.004**	2.92 (91)
**MR-6**	Covariates				
	Attended sessions	1.12	0.97–1.30	0.128	1.54 (91)
	BSI-18	1.12	0.85–1.47	0.413	0.82 (91)
	*R*^2^*_marg_ =* 0.094		*R*^2^*_cond_ =* 0.690		
**Family factors**					
	(Intercept)	0.79	0.33–1.87	0.588	−0.54 (94)
	Week	**1.04**	1.01–1.06	**<0.001**	3.40 (94)
**FCS**	Covariates				
	Attended sessions	1.00	0.87–1.14	0.983	−0.02 (94)
	BSI-18	0.78	0.60–1.01	0.059	−1.91 (94)
	*R*^2^*_marg_ =* 0.099		*R*^2^*_cond_ =* 0.722		
	(Intercept)	0.76	0.33–1.76	0.518	−0.65 (93)
	Week	**1.03**	1.00–1.06	0.031	2.19 (93)
**FB-K**	Covariates				
	Attended sessions	1.00	0.88–1.15	0.956	0.06 (93)
	BSI-18	0.91	0.71–1.17	0.473	−0.72 (93)
	*R*^2^*_marg_ =* 0.040		*R*^2^*_cond_ =* 0.627		
**Adolescent PG**					
	(Intercept)	4.89	2.46–9.71	<0.001	4.60 (89)
	Week	**0.97**	0.95–0.99	0.009	−2.69 (89)
**PIGDS**	Covariates				
	Attended sessions	**0.81**	0.73–0.90	**<0.001**	−3.88 (89)
	BSI-18	1.13	0.91–1.40	0.273	1.10 (89)
	*R*^2^*_marg_ =* 0.137		*R*^2^*_cond_ =* 0.875		

Notes: IRR = incidence rate ratio; df = degrees of freedom (based on Wald t-distribution approximation); an individual *p*-value ≤ 0.00833 according to Bonferroni correction can be interpreted as significant; PG = problematic gaming; PSS-4 = Perceived Stress Scale (4-item version); BSI-18 = Brief Symptom Inventory (18-item version); FSW = Parental Self-Efficacy Questionnaire; MR-6 = Media Rules Questionnaire (6 items); FCS = Family Communication Scale; FB-K = Family Questionnaire (*Familienbogen-Kurzversion*); PIGDS = Parental Internet Gaming Disorder Scale.

## 4. Discussion

Res@t-P is a newly developed group-therapy program for parents of adolescents with PG. It complements a group intervention for affected adolescents (Res@t-A) [33] and builds on a multifactorial treatment approach in line with the current state of research on the etiology of PG (e.g., [4,16]). To the best of our knowledge, no parental-training program specifically addressing the needs of affected parents is available at the moment. The presented study provides first results on the potential effectiveness of Res@t-P based on pilot data. Parental and family factors improved over the course of the 8-week intervention and the 6-week follow-up, together with a reduction in adolescent PG symptoms. Hence, Res@t-P has the potential to close a significant gap in the treatment of a new disorder in a critical age group with increasing public and healthcare concerns under the COVID-19 pandemic [54].

### 4.1. Parental Psychological Stress

Benefits of parental group programs are known, e.g., from treating ADHD in children and adolescents [55]. Since psychological stress is associated with mental health problems such as depression in the general population [56] and specifically in parents [57] that interfere with adolescents’ PG [21], it is addressed in Res@t module 7 by psychoeducation and group member interchange on stress-regulation strategies. A reduction in parental psychological stress could be found over the time course of the current study, with 22% of model variance being explained by time. A positive effect of short psychoeducation interventions on stress reduction has been meta-analytically shown [58]. Moreover, Res@t is accompanied by weekly mindfulness exercises and the participants are encouraged to include those in their daily life. Mindfulness interventions were effective for stress reduction based on a systematic review and meta-analysis with university students [59] and employees [60].

In addition, parents of the Res@t-P program qualitatively reported to feel relieved and supported by the exchange of experiences, worries, and needs associated with their child’s PG with other parents. In addition to the professional transfer of psychoeducative knowledge and skills, experiences as well as different views on and approaches to solving difficult situations can be shared by parents having undergone similar problems [61]. A perception of peer support results that might partially compensate deficits in parents’ social networks and reduces feelings of isolation and not being understood [61]. This in turn is associated with reduced stress [62]. Relatively small changes in stress perception per week might be explained by the heterogeneous sample and associated influences of contextual and personal factors, such as sex; age; educational, economical, and occupational background [39]; emotion regulation capabilities [63]; and mental distress [64], which was included as a covariate in the model.

### 4.2. Parenting Factors

#### 4.2.1. Parental Self-Efficacy

Although an increase in parental self-efficacy could be observed on a descriptive level, the corresponding *p*-value did not reach significance in the multilevel longitudinal model.

Parental self-efficacy refers to parents’ confidence about their impact on their child’s health and achievements and is associated with parental responsiveness, communication, parenting quality, and family role constructions [65]. Its increase serves as an important clinical target to promote children’s well-being, with largest effects for younger children [66].

In Res@t-P, parental self-efficacy is indirectly addressed by psychoeducation about PG (modules 1 and 2) and children’s development tasks (module 4), together with a reflection on parenting style and rules (modules 4–6). The adolescent age and the relatively short intervention and observation period might explain the non-significant effect. The covariate parental mental distress was negatively associated with parental self-efficacy in the model, which is in line with the literature and underlines the importance of therapeutic awareness of parental mental health [43,65].

#### 4.2.2. Media Rules

The implementation of and adherence to media-use associated rules significantly increased over the time of the intervention and follow-up. Those were specifically addressed in Res@t-P modules 5 and 6 by not only discussing specific parenting situations but also reflecting on parental attitudes and role models. Although being one of the main aspects in parental counseling, the literature on the impact of rules on the development of PG is partially contradictory [22,67,68]. Geurts and colleagues emphasize the importance of clear rules formulated in advance over reactive-impulsive rules in situations of parent-child conflicts [34]. Furthermore, a positive parent-child relationship influences the acceptance of rules [69]. Via media protocols of the parents’ own media use, their role functions are addressed throughout the training, in addition to role-specific contents of module 6 to increase self-awareness. The importance of parental role modeling and parental factors in the development of adolescents’ PG is supported by recent studies [34,35].

### 4.3. Family Factors

Family communication was addressed throughout Res@t-P by reflecting on validating and invalidating language during group discussions and individual verbal contributions. Moreover, psychoeducation and exercises to increase validating language and the use of “I” messages [70] took place in module 3. A significant increase in family communication quality could be observed over the course of the study. Thus, an important factor for a healthy interpersonal communication improved [71] that could be identified as a risk factor for PG development [72]. Communication is associated with parent-child relationship and family functioning [73]. Problematic family relationships are a risk factor for the development of PG [74] and relapse of addictive disorders [75]. They were indirectly addressed in modules 2 and 3 by externalizing the child’s problematic behavior as well as supporting positive views on the child and joint time. Family functioning increased during the training and follow-up, although the effect was not significant after Bonferroni correction of the *p*-value. This might be due to an indirect effect and the heterogeneous sample.

### 4.4. Adolescent PG Symptoms

Adolescents’ PG symptoms based on their parents’ ratings were negatively associated with the 14-week observation period, and the number of attended sessions served as a significant covariate. Due to the study design, the impact of Res@t-P on this finding cannot be interpretated causally. However, Res@t-P addresses important parental and family factors that were shown to affect PG symptoms [23,76]. In addition, research suggests decreased therapy dropouts in children with PG whose parents are involved in the treatment [29,30]. Thus, parental support by positive therapy expectations might increase adolescents’ therapy adherence [77] and facilitates PG symptom reduction.

### 4.5. Closing a Gap and Outlook

Current literature reports limited involvement of parents in an adolescent-focused therapy that is mainly based on psychoeducation and counseling [29,32,78,79]. Yet, empirical studies highlight the influence of parental and family factors on the development and maintenance of PG in children and adolescents [23]. An exception to no or only marginal parental involvement in adolescent PG treatment is an adaption of MDFT. MDFT has been established in the treatment of substance-use disorders in youths and involves sessions with the youth, the parents, and the family over six to nine months (e.g., [80]). However, it is a time-consuming treatment approach focusing on one affected adolescent and his or her family [81]. Given the shortage of appropriate treatment resources for PG in adolescence, the group setting of Res@t-P is a cost-efficient way to meet high demands [82,83].

In the PIPATIC program, parents are involved in four 45-min family sessions on psychoeducation, family communication, rules, and attachment [79]. Yet, although a non-randomized control-group study could find significant symptom reduction in adolescents with PG compared to baseline, no superiority of the PIPATIC program compared to classical CBT could be shown [28]. This might be due to the limited amount of time and less focus on specific parent’s demands.

The presented results strengthen the calls of international experts for the active involvement of parents in the therapy of children and adolescents with PG [33,84,85,86]. Accordingly, Res@t-P is conceptualized as part of the multifactorial Res@t program. Depending on the severity of the adolescent PG, adolescents’ comorbidities, and parental mental disorders, Res@t should be complemented by individual psychotherapy and psychopharmacotherapy (for the adolescent/parent, if appropriate), parental counseling, and individual family sessions. However, Res@t-P could also provide support for parents of affected children who refuse clinical attendance and treatment due to reduced introspection or symptom denial. This phenomenon can be observed in clinical practice [87] as a coping mechanism [88] leading to high false negative rates in PG questionnaires in adolescents [89].

Moreover, taking into account the psychosocial burden that an adolescent’s PG means for parents [37], a program for strengthening personal and parental skills, providing information, and encouraging the exchange of experiences within a parental peer group has the potential to enhance self-efficacy and problem solving strategies [61] as well as intervention cohesion by social support and increased motivation [90,91].

To reach a higher number of affected families, Res@t is currently translated into an online application following new treatment approaches [92]. Furthermore, comparisons between different cultural groups are aimed. Testing the program within a large RCT study is highly warranted and currently planned.

### 4.6. Limitations

The following limitations need to be considered: Firstly, the collection of self-report data is a common intervention research mechanism, although it is potentially biased by, e.g., third-party influence, social desirability, and memory recall problems. Parents were instructed to complete the questionnaires on their own and to their best knowledge. Moreover, data collection was supervised by experienced researchers. Secondly, the sample size is rather small. Yet, the number of participants is common for pilot studies and similar to other intervention studies in the field (e.g., [28,29,93,94,95]). Thirdly, no control group was included in the pre- and post-follow-up design. Since alternative therapy options for help-seeking parents are missing and resources are limited, they could not be assigned to alternative interventions or waiting groups for ethical reasons at this early stage of research. Thus, no randomization within a control-group design could be established. Although randomized-controlled intervention studies are urgently needed, they are still largely missing, especially with adolescent-parent target groups [26].

Fourthly, participating parents were recruited from different clinical settings, with their children being treated within outpatient or (partially) inpatient care. Hence, heterogenous adolescent severity of disease and treatment offers need to be considered. These might have increased outcome variability and reduced observed effects.

Fifthly, within the pilot study design, six different models were estimated based on the outcome variables of interest. Thus, a potential Type-I error needs to be considered and the Bonferroni-corrected *p*-value threshold was added. In addition, IRR were given with 95% CIs for better variable interpretation.

Lastly, the majority of participants (28/43) completed the training during the COVID-19 pandemic, with a potential impact on outcome measures. Accordingly, almost one-third of a large German representative sample of parents of adolescents reported a significant increase in psychological stress under social isolation, school closures, and broad restrictions [96]. This might have effected discontinuation or irregular attendance of the training. In addition, hygiene measures, such as wearing face masks throughout the sessions and keeping a 1.5 m distance, to ensure the continuity of the group might have had an impact on interactions and group dynamics.

## 5. Conclusions

Res@t-P is part of a multifactorial treatment approach and the first manualized program specifically addressing parents of adolescents with PG. The presented pilot data are promising regarding potential program effects on reducing psychological stress and also improving parenting and family factors. Moreover, an adolescent PG symptom reduction could be observed. Since no control group was included in the study design, the findings need to be treated with caution and no causality inferences can be made. However, given the early stage of research and the limited availability of intervention offers for affected families, Res@t-P has the potential to close a significant gap. Its evaluation within an RCT study is currently planned, as well as its adaptation to and application in different cultural contexts.

## Figures and Tables

**Table 1:** Sample characteristics.

Variable		Absolute Frequency (Relative Frequency in %)	Mean (SD)
*Sociodemographic measures*			
Parent			
	Mother	31 (72.1)	
	Father	12 (27.9)	
Age			
	All parents		48.72 (6.78)
	Mothers		46.74 (5.92)
	Fathers		53.83 (6.35)
Children			
	Number of children in a household		1.86 (0.77)
Relationship status			
	Married/Relationship	22 (51.2)	
	Single	5 (11.6)	
	Separated	15 (34.9)	
	Widowed	1 (2.3)	
Migration background			
	Yes	14 (32.6)	
	No	29 (67.4)	
Occupational status			
	Not working	7 (16.3)	
	Working part-time	14 (32.6)	
	Working fulltime	22 (51.2)	
School education			
	Lower school certificate (*Hauptschulabschluss*)	2 (4.7)	
	Secondary school certificate (*Realschulabschluss*)	11 (25.6)	
	University entry qualification (*Abitur*/*Fachhochschulreife*)	29 (67.4)	
	Other	1 (2.3)	
*Treatment measures*			
Participation			
	With the other parent	18 (41.9)	
	Alone	25 (58.1)	
Completers	Yes No	34 (79.07) 9 (20.93)	
Attended sessions			
	All parents		6.00 (1.95)
	Mothers		6.39 (1.80)
	Fathers		5.00 (2.05)
*Psychometric measures* Psychological factors			
PSS-4	Start of training (baseline) End of training (8 weeks after baseline) Follow-up (14 weeks after baseline)		7.4 (3.1) 6.9 (3.1) 6.0 (3.5)
BSI-18	Start of training (baseline) End of training (8 weeks after baseline) Follow-up (14 weeks after baseline)		7.9 (7.0) 8.0 (7.8) 5.9 (6.6)
Parenting factors			
FSW	Start of training (baseline) End of training (8 weeks after baseline) Follow-up (14 weeks after baseline)		41.2 (4.4) 41.5 (5.4) 42.6 (5.1)
MR-6	Start of training (baseline) End of training (8 weeks after baseline) Follow-up (14 weeks after baseline)		7.4 (4.6) 9.4 (5.0) 9.6 (4.5)
Family factors			
FB-K	Start of training (baseline) End of training (8 weeks after baseline) Follow-up (14 weeks after baseline)		32.7 (6.7) 36.5 (7.0) 35.7 (8.5)
FCS	Start of training (baseline) End of training (8 weeks after baseline) Follow-up (14 weeks after baseline)		25.3 (6.0) 29.1 (6.4) 28.8 (7.3)
Adolescent PG			
PIGDS	Start of training (baseline) End of training (8 weeks after baseline) Follow-up (14 weeks after baseline)		8.2 (1.8) 6.9 (2.7) 6.9 (2.9)

Notes: SD = standard deviation; PG = problematic gaming; PSS-4 = Perceived Stress Scale (4-item version); BSI-18 = Brief Symptom Inventory (18-item version); FSW = Parental Self-Efficacy Questionnaire; MR-6 = Media Rules Questionnaire (6 items); FCS = Family Communication Scale; FB-K = Family Questionnaire (*Familienbogen-Kurzversion*); PIGDS = Parental Internet Gaming Disorder Scale.

## Data Availability

The data presented in this study are available on reasonable request from the corresponding author.
